# Antioxidant Defenses in the Brains of Bats during Hibernation

**DOI:** 10.1371/journal.pone.0152135

**Published:** 2016-03-24

**Authors:** Qiuyuan Yin, Hanxiao Ge, Chen-Chong Liao, Di Liu, Shuyi Zhang, Yi-Hsuan Pan

**Affiliations:** 1 Laboratory of Molecular Ecology and Evolution, Shanghai Engineering Research Center of Molecular Therapeutics and New Drug Development, East China Normal University, Shanghai, China; 2 Proteomic Research Center, National Yang-Ming University, Taipei, Taiwan; 3 State Key Laboratory of Estuarine and Coastal Research, East China Normal University, Shanghai, China; 4 College of Animal Science and Veterinary Medicine, Shenyang Agricultural University, Shenyang, China; 5 Laboratory of Molecular Ecology and Evolution, School of Life Sciences, East China Normal University, Shanghai, China; Universidade de Brasília, BRAZIL

## Abstract

Hibernation is a strategy used by some mammals to survive a cold winter. Small hibernating mammals, such as squirrels and hamsters, use species- and tissue-specific antioxidant defenses to cope with oxidative insults during hibernation. Little is known about antioxidant responses and their regulatory mechanisms in hibernating bats. We found that the total level of reactive oxygen species (ROS) and reactive nitrogen species (RNS) in the brain of each of the two distantly related hibernating bats *M*. *ricketti* and *R*. *ferrumequinum* at arousal was lower than that at torpid or active state. We also found that the levels of malondialdehyde (product of lipid peroxidation) of the two hibernating species of bats were significantly lower than those of non-hibernating bats *R*. *leschenaultia* and *C*. *sphinx*. This observation suggests that bats maintain a basal level of ROS/RNS that does no harm to the brain during hibernation. Results of Western blotting showed that hibernating bats expressed higher amounts of antioxidant proteins than non-hibernating bats and that *M*. *ricketti* bats upregulated the expression of some enzymes to overcome oxidative stresses, such as superoxide dismutase, glutathione reductase, and catalase. In contrast, *R*. *ferrumequinum* bats maintained a relatively high level of superoxide dismutase 2, glutathione reductase, and thioredoxin-2 throughout the three different states of hibernation cycles. The levels of glutathione (GSH) were higher in *M*. *ricketti* bats than in *R*. *ferrumequinum* bats and were significantly elevated in *R*. *ferrumequinum* bats after torpor. These data suggest that *M*. *ricketti* bats use mainly antioxidant enzymes and *R*. *ferrumequinum* bats rely on both enzymes and low molecular weight antioxidants (e.g., glutathione) to avoid oxidative stresses during arousal. Furthermore, Nrf2 and FOXOs play major roles in the regulation of antioxidant defenses in the brains of bats during hibernation. Our study revealed strategies used by bats against oxidative insults during hibernation.

## Introduction

During hibernation, small hibernating mammals (e.g., bats, hamsters, and ground squirrels) go through a repeated torpor-arousal cycle characterized by an alternate physiological state of heterothermy and homeothermy. Usually, a torpor bout persists for several days or weeks, and an arousal bout lasts less than a day. During torpor, many physiological functions such as body temperature, heart contraction, mitochondrial respiration, oxygen consumption, and blood flow are reduced to an extreme low level that can be lethal to many homeotherms [1]. However, these functions are promptly restored to the basic level upon arousal [2]. Each cycle is precisely controlled to meet the overall energy requirement throughout hibernation [1–3].

The torpor-arousal cycles of small mammals have features similar to a repeated process of ischemia and reperfusion [4, 5]. During reperfusion, a quick restoration of blood flow accompanied by an increased mitochondrial respiration and oxygen usage results in elevated generation of reactive oxygen species (ROS) in mammals [6, 7]. Excessive production or accumulation of ROS (e.g., H_2_O_2_ and ROO∙) and reactive nitrogen species (e.g., NO and ONOO∙^-^) may cause oxidative damage that leading to ageing [8] and diseases such as Parkinson’s disease, Alzheimer’s disease, diabetes, and stroke [9]. However, no ischemia-reperfusion injury is found in the brains of ground squirrels during a prolonged period of hibernation [4, 10]. The long-term memory of bats also remains intact during hibernation [11]. Small hibernators have, therefore, been used to study ischemic tolerance in order to protect humans against cerebral ischemic damages and neurodegenerative diseases [12–14].

Brain is the most hypoxia-sensitive organ and is vulnerable to oxidative damage. Different brain regions (e.g., hippocampus, lateral ventricles, and hypothalamus) act cooperatively to regulate hibernation processes [15]. It is known that hibernating mammals have antioxidant defense systems against oxidative stress [13, 15–17] and the amounts of antioxidant proteins, low molecular weight antioxidants (LMWAs), and malondialdehyde (MDA) vary among different stages of torpor-arousal cycles [14, 17–21], implying that the antioxidant defense system is reorganized in response to the redox imbalance during hibernation.

An antioxidant defense system generally consists of antioxidant proteins and LMWAs (e.g., glutathione, urate, and ascorbate) [22], which effectively scavenges free radicals (e.g., ROS and RNS) to maintain redox homeostasis of the cell (S1 Fig) [23–33]. Several transcription factors, such as nuclear factor-erythroid 2-related factor-2 (Nrf2) and forkhead box O proteins (FOXOs), including FOXO1, FOXO3a, and FOXO4, are activated by ROS [34, 35]. These transcription factors induce the expression of numerous antioxidant proteins [35, 36]. Under normal conditions, Nrf2 is retained in the cytoplasm by its inhibitor Kelch-like ECH-associated protein 1 (Keap1). This complex can be directed towards degradation by ubiquitination [37]. Increased level of ROS can cause release of Nrf2 from Keap1. The released Nrf2 is translocated into the nucleus to interact with other transcription factors (e.g., Mafs and CBP/p300) to stimulate the expression of its target genes [37]. Activation of Nrf2 is also mediated by post-translational modifications such as phosphorylation [38], acetylation [39], and sumoylation [40]. Although increased expression of Nrf2 and FOXO1A have been detected in mammalian hibernators [5, 41], the mechanisms of expression control of antioxidant proteins in hibernating bats remain unknown.

Bats account for a quarter of mammalian species. The phylogenic relationship among different bat species is well defined [42]. Microbats (e.g., *Myotis ricketti* in Yangochiroptera and *Rhinolophus ferrumequinum* in Yinpterochiroptera) are distributed in temperate or cold latitudes. They hibernate, eat mainly insects, and have a longevity of approximately 35 years [43–46], whereas megabats belong only to Yinpterochiroptera (e.g., *Cynopterus sphinx* and *Rousettus leschenaultia*) reside in tropical latitude. Most megabats do not hibernate; they consume flowers and fruits and have a lifespan of less than 15 years (S1 Table) [47]. Previous studies suggest that a longer lifespan of hibernating bats is due, in part, to their low production of free radicals [44, 48] and that the induction of antioxidant defenses is an adaptive response to hibernation [19]. However, the total level of ROS/RNS in the brain of any hibernating mammalian species has not been determined. The response and regulation of antioxidant defense system during bat hibernation are largely unclear.

We hypothesize that hibernating bats adjust their antioxidant defense in order to prevent oxidative damage to the brain in various stages of hibernation. To test this hypothesis, we determined the total level of ROS/RNS in the brains of two distantly related hibernating bats, *M*. *ricketti* and *R*. *ferrumequinum* at different hibernation states, as well as that of non-hibernating *R*. *leschenaultia* and *C*. *sphinx* bats, rats, and mice. The amounts of sulfhydryl glutathione, glutathione disulfide, and MDA in the brains of these species were also measured. The expression levels of antioxidant proteins (superoxide dismutase, glutathione reductase, glutathione peroxidase, catalase, peroxiredoxins, NADPH quinone oxidoreductase 1, DJ-1, and thioredoxin-2) of bats at different states were determined. Moreover, we explored the molecular mechanisms for the regulation of antioxidant responses in bat brain during hibernation.

## Material and Methods

### Ethics Statement

The field studies in this research did not involve endangered or protected species. All animal experiments were strictly followed the Guidelines and Regulations for the Administration of Laboratory Animals (Decree No. 2, the State Science and Technology Commission of the People's Republic of China, November 14, 1998) and were approved by the Animal Ethics Committee of East China Normal University (approval number AR2012/03001).

### Animal and tissue acquisition

Hibernating bats *M*. *ricketti* (n = 12) and *R*. *ferrumequinum* (n = 12) were captured from Fangshan Cave (39°48'N, 115°42'E) in Beijing and Fish Cave (30°20'N, 117°50'E), in Anhui Province, China, respectively. Four torpid bats from each species were sacrificed on site upon capture, and four bats from each species were spontaneously aroused upon capture and sacrificed 2 hours after arousal. The remaining bats were scarified 24 hours after arousal from torpor in our laboratory where the room temperature was 27°C. Active bats (24 hours after arousal) had higher rectal temperatures and fasting blood glucose levels than bats at torpor or 2 hours after arousal (S2 Table). They also quickly responded to human disturbance. Non-hibernating bats *R*. *leschenaultia* (n = 4) and *C*. *sphinx* (n = 4) were sacrificed immediately after they were captured from Jinlun Cave of Mashan County (23°55'N, 108°26'E) in Guangxi Province and Haikou Park (20°02'N, 110°20'E) in Hainan Province, China, respectively. No water and food were given to these bats during fasting. The whole brain of each bat was rapidly removed and snap frozen in liquid nitrogen. Sprague-Dawley rats (*Rattus norvegicus*, n = 4) and Kunming mice (*Mus musculus*, n = 4), maintained in a 12-hour dark-light cycle at 26°C, were obtained from Sino-Britsh Sippr/BK Lab Animal Ltd (Shanghai, China) and sacrificed upon arrival in our laboratory. The whole brains were transferred to -80°C until used.

All animals including bats, rats, and mice used in this study were adult males. They were sacrificed by cervical dislocation to minimize pain and suffering. Their rectal temperatures, body weights, and fasting blood glucose concentrations are listed in S2 Table.

### Quantification of ROS and RNS

The OxiSelect^™^ *In Vitro* ROS/RNS Assay Kit (Cell Biolabs Inc., San Diego, CA) containing a specific ROS/RNS probe, dichlorodihydrofluorescein DiOxyQ (DCFH-DiOxyQ), was used to measure the total amounts of reactive oxygen and nitrogen species (referred to as ROS/RNS hereafter), including hydrogen peroxide, peroxyl radical, nitric oxide, and peroxynitrite anion. In this assay, the probe was oxidized by ROS/RNS to generate a fluorescent product dichlorofluorescein (DCF). The assay was performed according to manufacturer’s instructions. Briefly, the mashed brain tissue (50 mg) was homogenized in 1 ml PBS (137 mM, NaCl, 2.7 mM KCl, 2 mM KH_2_PO_4_, and 10 mM Na_2_HPO_4_) at 4°C with a Precellys^®^ 24 homogenizer (Bertin technologies, France). After the homogenate was centrifuged at 10000 xg, 4°C for 5 min, the clarified supernatant was collected and diluted 10 fold. An aliquot (50 μl) of the diluted supernatant was incubated with 50 μl of the catalyst reagent of the assay kit for 5 min and then with 100 μl of DCFH-DiOxyQ solution in the dark for 30 min at room temperature. The fluorescence of DCF thus generated was measured with a SynergyT^M^ HT spectrophotometer (Biotek, Winooski, VT) at excitation/emission wavelengths 480/530 nm. The concentration of ROS/RNS was determined fluorometrically against the DCF standards.

### TBARS Assay for Lipid peroxidation

Since lipid peroxidation forms malondialdehyde (MDA) and 4-hydroxynonenal (4-HNE), the levels of MDA or 4-HNE are a measure of oxidative damage. MDA readily reacts with thiobarbituric acid (TBA) to generate the MDA-TBA adduct (one type of thiobarbituric acid reactive substances, TBARS), which can be quantified colorimetrically. The levels of MDA in brain tissue samples were measured by the lipid peroxidation MDA Assay Kit (Beyotime Institute of Biotechnology, China). Briefly, the clarified supernatant derived from 100 μl of a brain homogenate was mixed with the assay reagent (200 μl) containing TBA and butylated hydroxytoluene (BHT); the latter reduces the artefactual formation of lipid peroxides. The mixture was heated at 100°C for 15 min. After cooling, the absorbance of the mixture was measured at 532 nm. MDA concentration in a sample was determined by comparing its OD_532_ value against those of the MDA standards.

### Immunoblotting

The mashed brain tissue (100 mg) was homogenized in 2 ml lysis buffer (10% glycerol, 2% SDS, 3.12 mM EDTA, 1.25% β-mercaptoethanol, 25 mM Tris-HCl, pH 6.8) with a Precellys^®^ 24 grinder (Bertin technologies, France). After centrifugation at 12,000 xg, 4°C for 15 min, the supernatant was collected, heated at 100°C for 10 min, and then stored at -80°C until used. Protein concentration of each sample was determined as previously described [49]. Equal amount of proteins of various samples were separated by SDS-PAGE and then transferred onto 0.2 μm PVDF membranes (Millipore, USA). Each protein-bearing membrane was incubated in blocking solution (5% skim milk and 1% BSA) at 4°C for 12h before the primary antibody was applied. Primary antibodies against SOD1, SOD2, GSR, GPX1, CAT, NQO1, DJ-1 (PARK7), TRX2, PRDX1, PRDX3, Nrf2, Nrf2 (phospho S40), KEAP1, FOXO1 (FKHR), and FOXO3A are listed in S3 Table. These antibodies were chosen for their capability to react with conserved epitopes of the protein across diverse species. The antigen-antibody reactions on blots were visualized using the Immobilon^™^ Western Chemiluminescence HRP substrate kit (Millipore, USA). Images were captured with ImageQuant^™^LAS-4000 (Amersham Biosciences, USA). The target bands were quantified by ImageQuant^™^TL software (version 7.0, Amersham Biosciences, USA). Ponceau S stained blots were used to evaluate sample loading [50]. The intensity of each band on an immunoblot was normalized to that of the corresponding Ponceau S stained band.

### Determination of GSSG/GSH ratio

The levels of total glutathione including disulfide (GSSG) and reduced (GSH) forms were determined by the colorimetric GSSG/GSH assay kits (Beyotime Institute of Biotechnology, China). In this assay, GSSG is reduced by glutathione reductase in the presence of NADPH to become GSH; both the preexisting and converted GSH molecules then reduce 5, 5’-dithiobis-2-nitrobenzonic acid (DNTB) to 2-nitriobenzonic acid (TNB), which is measured spectrophotometrically at 412 nm. Briefly, a mashed brain tissue (50 mg) was homogenized in 1 ml PBS, and the homogenate was centrifuged at 10000 xg, 4°C for 5 min. The supernatant obtained was diluted 8 fold with metaphosphoric acid. After an incubation at 4°C for 10 min, the precipitated proteins were removed by centrifugation at 10,000 xg, 4°C for 10 min. The resulting supernatant (10 μl) was incubated with 200 μl of the assay reagent containing glutathione reductase and NADPH for 5 min at 25°C, and the amounts of TNB thus formed were determined by measuring OD_412_. For determination of GSSG levels, the supernatant of the sample was pre-treated with 2-vinylpyridine to mask the SH group of GSH, so that GSH could not be converted to GSSH. Therefore, only GSSG in the sample was measured. The level of GSH was determined by subtracting the amount of GSSG from that of total glutathione in the sample.

### IPA prediction of transcription regulation network

Accession numbers (from UniProt or KEGG) and changes in the levels of antioxidant proteins, glutathione, malondialdehyde, and ROS/RNS are listed in S4 Table. The interaction and regulation network of these molecules were analyzed by the Ingenuity^®^ Pathway Analysis (IPA) software (version 23814503) according to their functions, expression levels, and interactions as previously described [51]. The significance (*P* value of overlap) was determined by the Fisher’s exact test. A *P* value < 0.001 was considered significant.

### Statistical analysis

Three repeats of each experiment were conducted. Data were presented as mean ± SD and summarized as box plots showing the mean, 25/75 percentiles (box), and 10/90 percentiles (bars). A *P* value < 0.05 was considered significant in a group comparison by One Way Analysis of Variance (Holm-Sidak method).

## Results

### Reduced ROS/RNS levels in the brains of hibernating bats at arousal

**Fig 1:**
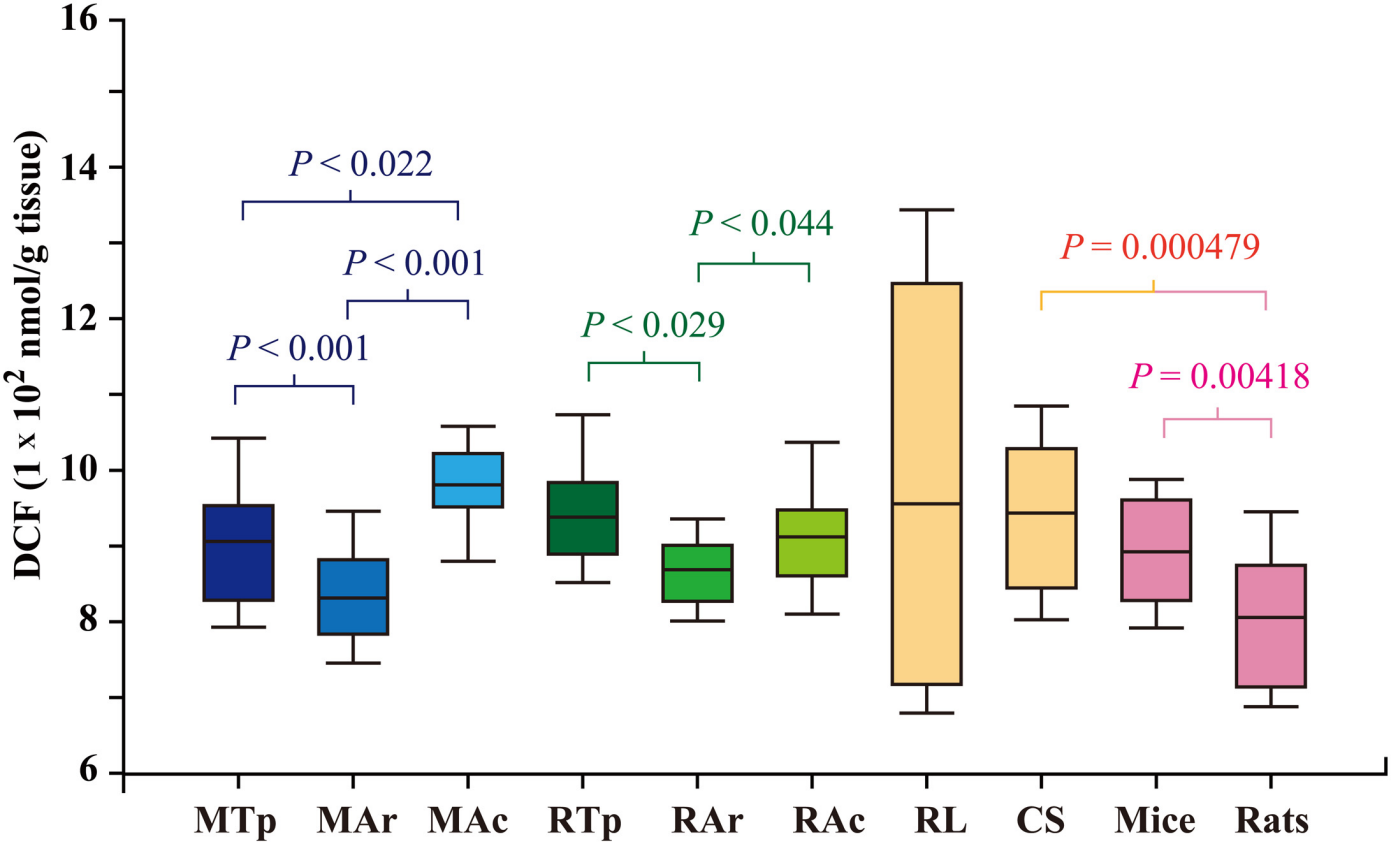
Total ROS/RNS levels in brain tissue. Total ROS/RNS levels are represented by DCF contents. Y-axis represents total amounts of DCF (1 X 10^2^ nmol/g tissue). *M*. *ricketti* bats at torpor, arousal, and active states are indicated as MTp, MAr, and MAc, respectively. *R*. *ferrumequinum* bats at torpor, arousal, and active states are indicated as RTp, RAr, and RAc, respectively. Brain ROS/RNS levels of *R*. *leschenaultia* (RL), *C*. *sphinx* (CS), mice, and rats are also shown. Data are presented as box plots that show the mean, 25/75 percentiles (box), and 10/90 percentiles (bars). A *P* value < 0.05 is considered significant.

The ROS/RNS levels (DCF nmol/g of brain tissue) of the following species were determined: two distantly related species of hibernating bats (*M*. *ricketti* and *R*. *ferrumequinum*) at torpid (Tp), arousal (Ar), and active (Ac) states and two different species of non-hibernating bats (*R*. *leschenaultia* and *C*. *sphinx*) (Fig 1). As outgroup members for comparison, ROS/RNS levels in the brains of rats and mice were also determined. For both *M*. *ricketti* and *R*. *ferrumequinum* bats, the mean ROS/RNS levels were the lowest at arousal (815.54 ± 46.57 and 869.55 ± 49.56 nmol/g, respectively). ROS/RNS levels in the brain of *M*. *ricketti* bats during the active state were significantly higher than those during the torpor state (981.16 ± 58.00 vs. 915.82 ± 73.71 nmol/g, respectively). For *R*. *ferrumequinum* bats, ROS/RNS levels during the active state were slightly lower than those during the torpor state (911.92 ± 71.33 vs. 924.99 ± 67.21 nmol/g); however, this difference was not statistically significant (*P* = 0.548). For non-hibernating *R*. *leschenaultia* bats, ROS/RNS levels varied widely among individual bats, with a mean level of 956.04 ± 274.90 nmol/g. For *C*. *sphinx*, another non-hibernating species, the mean ROS/RNS level was 943.64 ± 104.03 nmol/g, similar to that of *R*. *leschenaultia*, but the variation in levels among individual bats was not as big. For outgroup members, the mean ROS/RNS level of rats (800.66 ± 76.17 nmol/g) was lower than that of mice (892.22 ± 74.74 nmol/g).

### Lower level of MDA in hibernating bats

**Fig 2:**
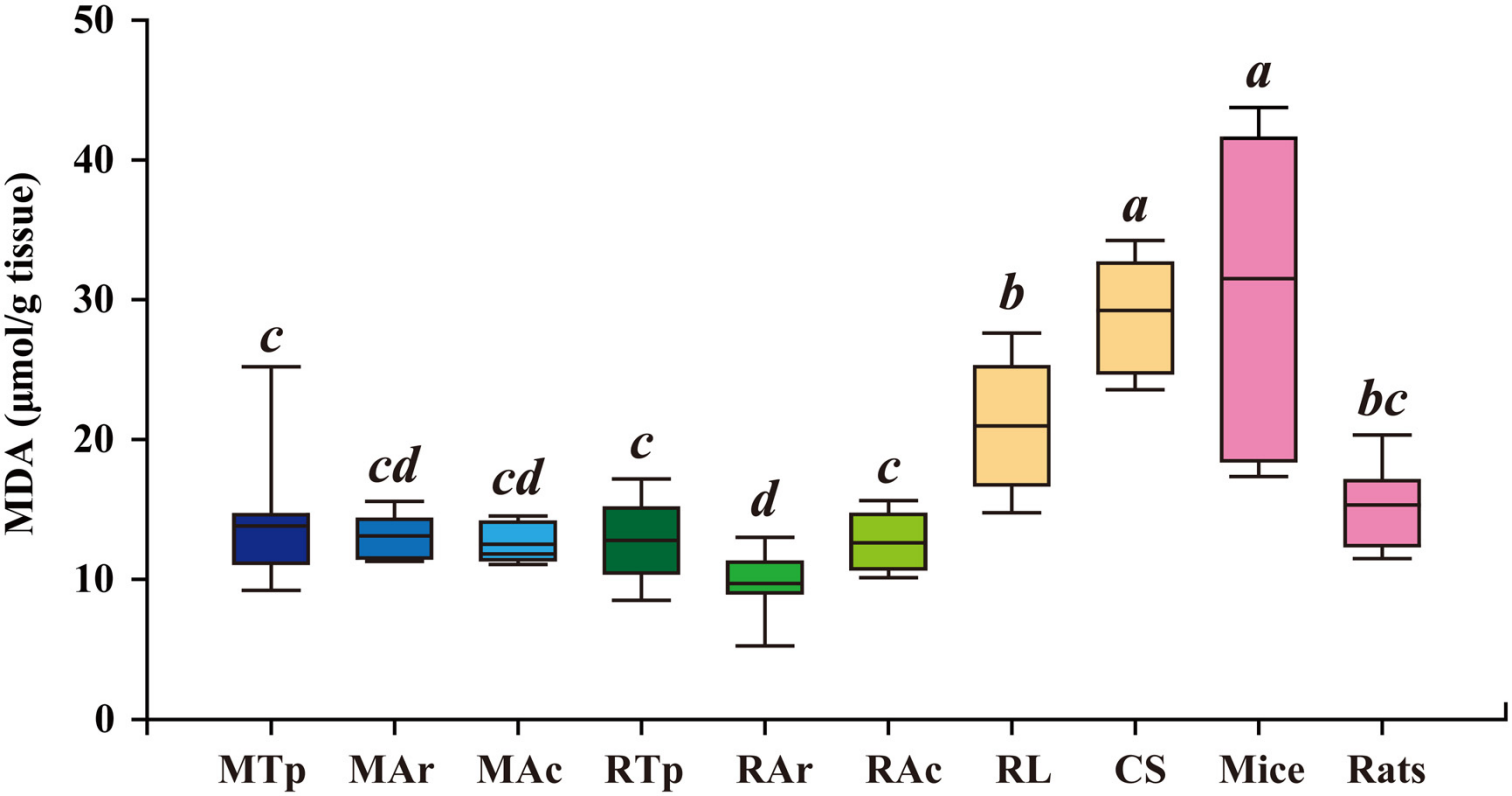
Malondialdehyde (MDA) levels in brain tissue. Y-axis represents MDA levels (μmol/g tissue). *M*. *ricketti* bats at torpor, arousal, and active states are indicated as MTp, MAr, and MAc, respectively. *R*. *ferrumequinum* bats at torpor, arousal, and active states are indicated as RTp, RAr, and RAc, respectively. Brain MDA levels of *R*. *leschenaultia* (RL), *C*. *sphinx* (CS), mice, and rats are also shown. Data are presented as box plots that show the mean, 25/75 percentiles (box), and 10/90 percentiles (bars). Values of group means are represented by italicized letters and are in the order of *a* > *b* > *bc* > *c* > *cd* > *d*. The difference in value is significant (*P* < 0.05) between different letters.

Since ROS can degrade polyunsaturated lipids to form malondialdehyde (MDA) that causes toxic stress in cells, MDA levels in the brains of hibernating (*M*. *ricketti* and *R*. *ferrumequinum*) and non-hibernating (*R*. *leschenaultia* and *C*. *sphinx*) bats, as well as mice and rats, were measured. The brain MDA levels were determined to be 13.81 ± 4.91 μmol/g, 13.10 ± 1.69 μmol/g, and 12.51 ± 1.39 μmol/g for torpid, arousal, and active *M*. *ricketti* bats, respectively; there was no significant difference among these levels. For *R*. *ferrumequinum* bats, the mean brain MDA level (9.72 ± 2.44 μmol/g) at arousal was lower than that at torpid (12.79 ± 3.15 μmol/g) or active (12.61 ± 1.93 μmol/g) state (Fig 2). Brain MDA levels of hibernating bats at any state were significantly lower than those of non-hibernating species, including *R*. *leschenaultia* bats (20.96 ± 4.69 μmol/g), *C*. *sphinx* bats (28.01 ± 4.35 μmol/g), and mice (31.51 ± 11.32 μmol/g). Rats had a significantly lower brain MDA level (15.33 ± 2.96 μmol/g) than mice and non-hibernating bats.

### Increased expression of antioxidant proteins in *M*. *ricketti* bats after arousal

**Fig 3:**
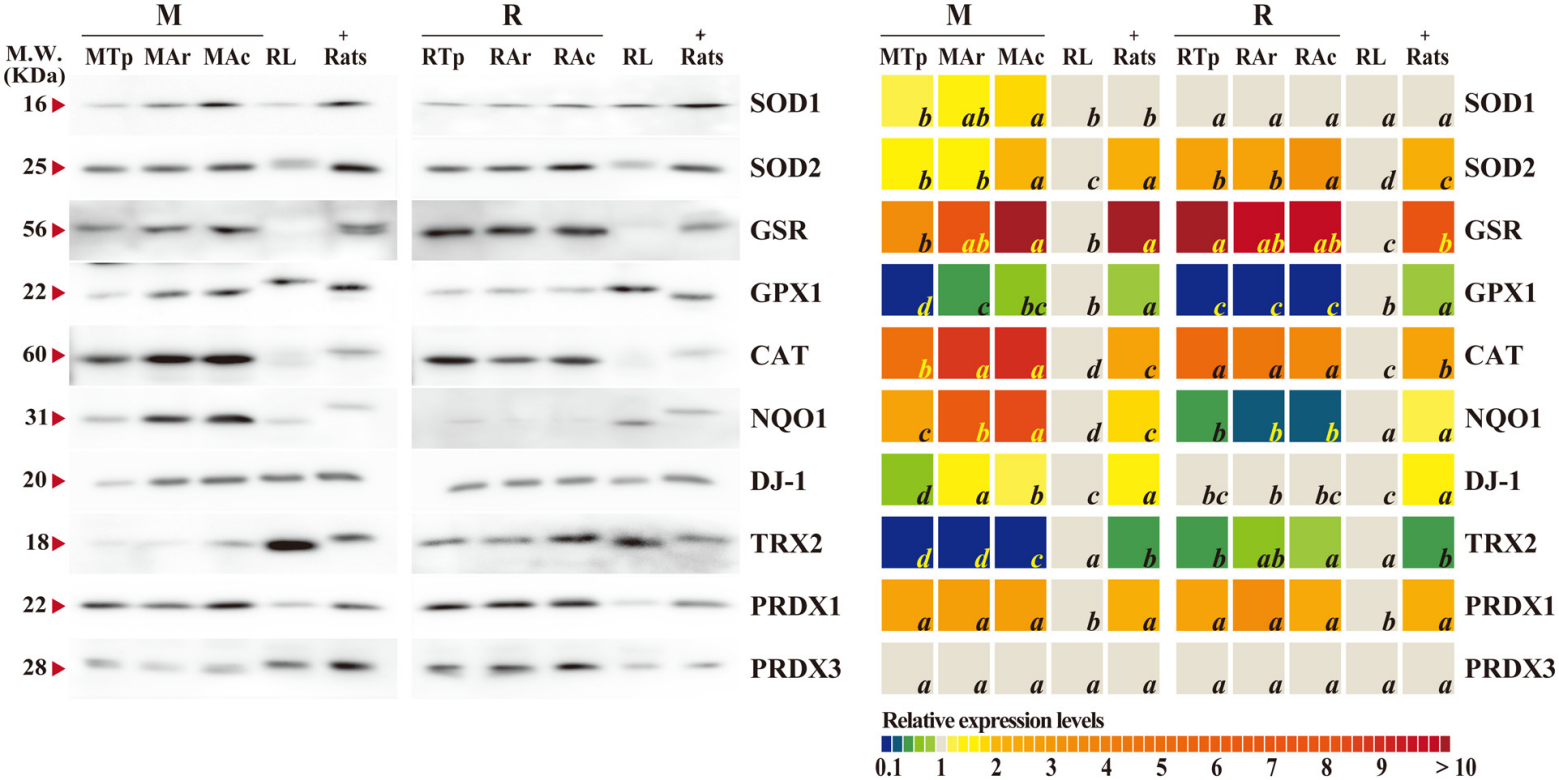
Relative expression levels of antioxidant enzymes. Left panel shows Western blot band intensity of SOD1, SOD2, GSR, GPX1, CAT, NQO1, DJ-1, TRX2, PRDX1, and PRDX3 in *M*. *ricketti* bats (M), *R*. *ferrumequinum* bats (R), non-hibernating *R*. *leschenaultia* bats (RL), and rats. The bands of rats are used as positive controls (+) for antibody reaction. MTp, MAr, and MAc represent *M*. *ricketti* bats at the torpor, arousal, and active states, respectively. RTp, RAr, and RAc indicate *R*. *ferrumequinum* bats at their torpor, arousal, and active states, respectively. Predicted molecular weights (KDa) are denoted by red arrowheads. Right panel is a heat map showing relative expression levels of antioxidants from 0.1 to >10. The band density of each detectable protein of RL is set as 1 and is shown in gray. The relative expression level of a protein is calculated by dividing its band density value by that of RL. Values of group means are represented by italicized letters and are in the order of *a* > *ab* > *b* > *bc* > *c* > *d*. The difference in value is significant (*P* < 0.05) between different letters.

To investigate antioxidant defenses, the levels of ten antioxidant proteins in the brain tissue samples were determined. Hibernating bats at torpid, arousal, and active states and non-hibernating bats, and rats were examined (Fig 3, S1 Fig and S5 Table). Both SOD1 and SOD2 levels were found to be significantly higher in the brains of active than in torpid *M*. *ricketti* bats. SOD2 levels were also higher in active than in torpid *R*. *ferrumequinum* bats (Fig 3). The expression of GSR in *M*. *ricketti* bats was increased after arousal, and the level was significantly higher in active than in torpid bats. GSR levels in *R*. *ferrumequinum* bats remained high at all three states (Fig 3). In *M*. *ricketti* bats, the amount of GPX1 in arousal or active state was higher than that in torpid state. No significant difference in GPX1 expression among the three states was found in *R*. *ferrumequinum* bats (Fig 3). An elevated expression of CAT and NQO1 was detected in *M*. *ricketti* bats after arousal (Fig 3), but no significant difference in the expression of these two proteins among the three different states was detected in *R*. *ferrumequinum* bats (Fig 3). The expression levels of DJ-1 in *M*. *ricketti* bats at arousal or active state were higher than that at torpid state, but were about the same among the three states in *R*. *ferrumequinum* bats (Fig 3). TRX2 was expressed at very low levels in all three states of *M*. *ricketti* bats. In *R*. *ferrumequinum* bats, TRX2 expression was higher in active than in torpid or arousal state (Fig 3 and S1 Fig). PRDX1 and PRDX3 showed no difference in expression in any hibernating bats at any states (Fig 3).

The expressions of SOD2, GSR, CAT, and PRDX1 were found to be higher in active hibernating bats than in non-hibernating bats (Fig 3). In contrast, the levels of GPX1 in active *R*. *ferrumequinum* bats were lower than those of *R*. *leschenaultia* bats, and the TRX2 levels in active *M*. *ricketti* bats were lower than those of *R*. *leschenaultia* bats (Fig 3). The differences in expression of these proteins between active and non-hibernating bats were similar to those between non-hibernating bats and rats (Fig 3).

### Elevated level of total GSH in active bats

**Fig 4:**
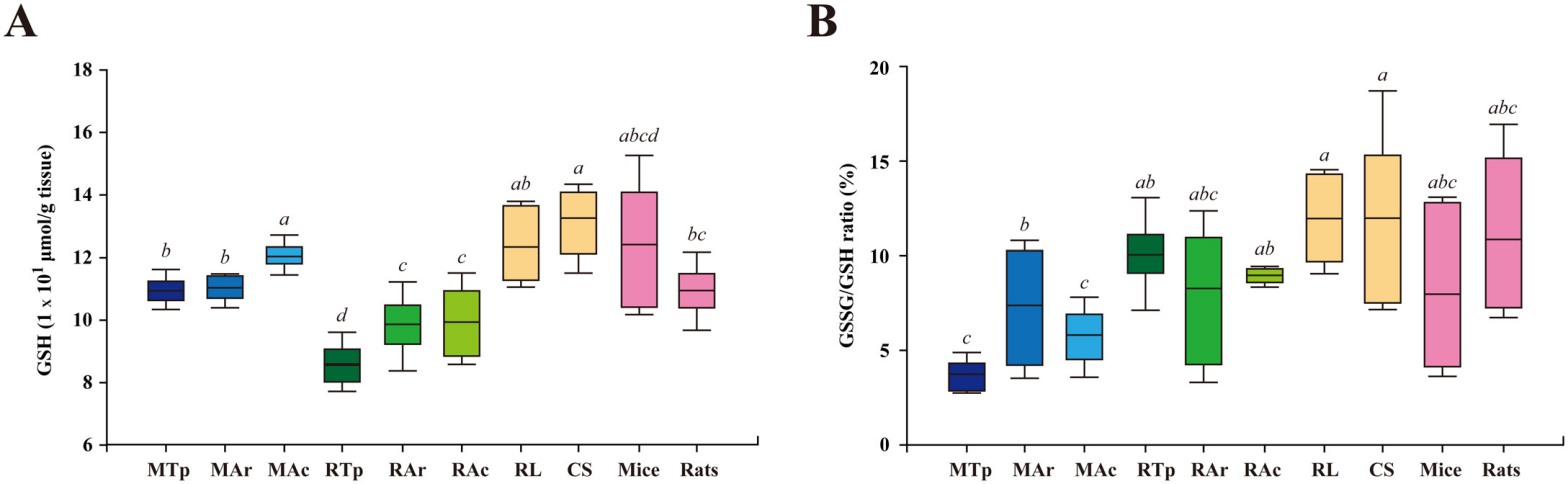
Total levels of GSH and the ratio of GSSG/GSH. **(A)** Total amounts of GSH (μmol/g tissue) and **(B)** GSSG/GSH ratios of the following were determined: *M*. *ricketti* bats at torpor (MTp), arousal (MAr), and active states (MAc); *R*. *ferrumequinum* bats at torpor (RTp), arousal (RAr), and active states (RAc), non-hibernating bats *R*. *leschenaultia* (RL) and *C*. *sphinx* (CS), mice, and rats. Box plots show the mean, 25/75 percentiles (box), and 10/90 percentiles (bars). Values of group means are represented by italicized letters and are in order of *a* > *ab* > *abc* > *abcd* > *b* > *bc* > *c* > *cd* > *d*. The difference in value is significant (*P* < 0.05) between different letters.

Because GSH scavenges free radicals [30, 31], its levels in the brains of hibernating and non-hibernating bats, as well as mice and rats were determined (Fig 4A). In *M*. *ricketti* bats, GSH levels were higher at active (120.40 ± 3.85 μmol/g) than at torpid (109.44 ± 4.03 μmol/g) or arousal (110.35 ± 4.16 μmol/g) state. GSH levels in *R*. *ferrumequinum* bats were 85.96 ± 6.47 μmol/g at torpor, 98.68 ± 9.35 μmol/g at arousal, and 99.40 ± 10.55 μmol/g at active state (Fig 4A). At any of the three states, GSH levels were higher in *M*. *ricketti* bats than in *R*. *ferrumequinum* bats.

GSH levels in non-hibernating *R*. *leschenaultia* (123.33 ± 11.69 μmol/g) and *C*. *sphinx* bats (132.58 ± 10.46 μmol/g) were significantly higher than in hibernating *R*. *ferrumequinum* bats at any states (Fig 4A). Non-hibernating *C*. *sphinx* bats also had a higher level of GSH than torpid or arousal *M*. *ricketti* bats. Rats (109.52 ± 7.91 μmol/g) had a significant lower GSH level than non-hibernating bats. GSH levels varied widely among individual mice with a mean level of 119.00 ± 19.37 μmol/g, similar to that of *C*. *sphinx* bats.

To understand the redox status, the ratio of glutathione disulfide (GSSG) to GSH was determined. *M*. *ricketti* bats were found to have a higher GSSG to GSH ratio (7.4% ± 2.9%) at arousal than at torpor (3.7% ± 0.8%) or active (5.8% ± 1.5%) state (Fig 4B). GSSG to GSH ratios of torpid, arousal, and active *R*. *ferrumequinum* bats were 10.1% ± 1.8%, 8.3% ± 3.2%, and 9% ± 0.4%, respectively; no significant changes were detected among these three states (Fig 4B). The GSSG to GSH ratios of torpid and active *R*. *ferrumequinum* bats were significantly higher than those of torpid and active *M*. *ricketti* bats (Fig 4B).

GSSG to GSH ratios of non-hibernating bats (*R*. *leschenaultia*, 12% ± 2.2%; *C*. *sphinx* bats, 12% ± 4.2%) were significantly higher than those of hibernating *M*. *ricketti* bats at any of the three states (Fig 4B). The GSSG to GSH ratios varied widely among individual mice, rats, and both species of hibernating bats at arousal (Fig 4B).

### Transcriptional regulation of antioxidant responses in hibernating bats

**Fig 5:**
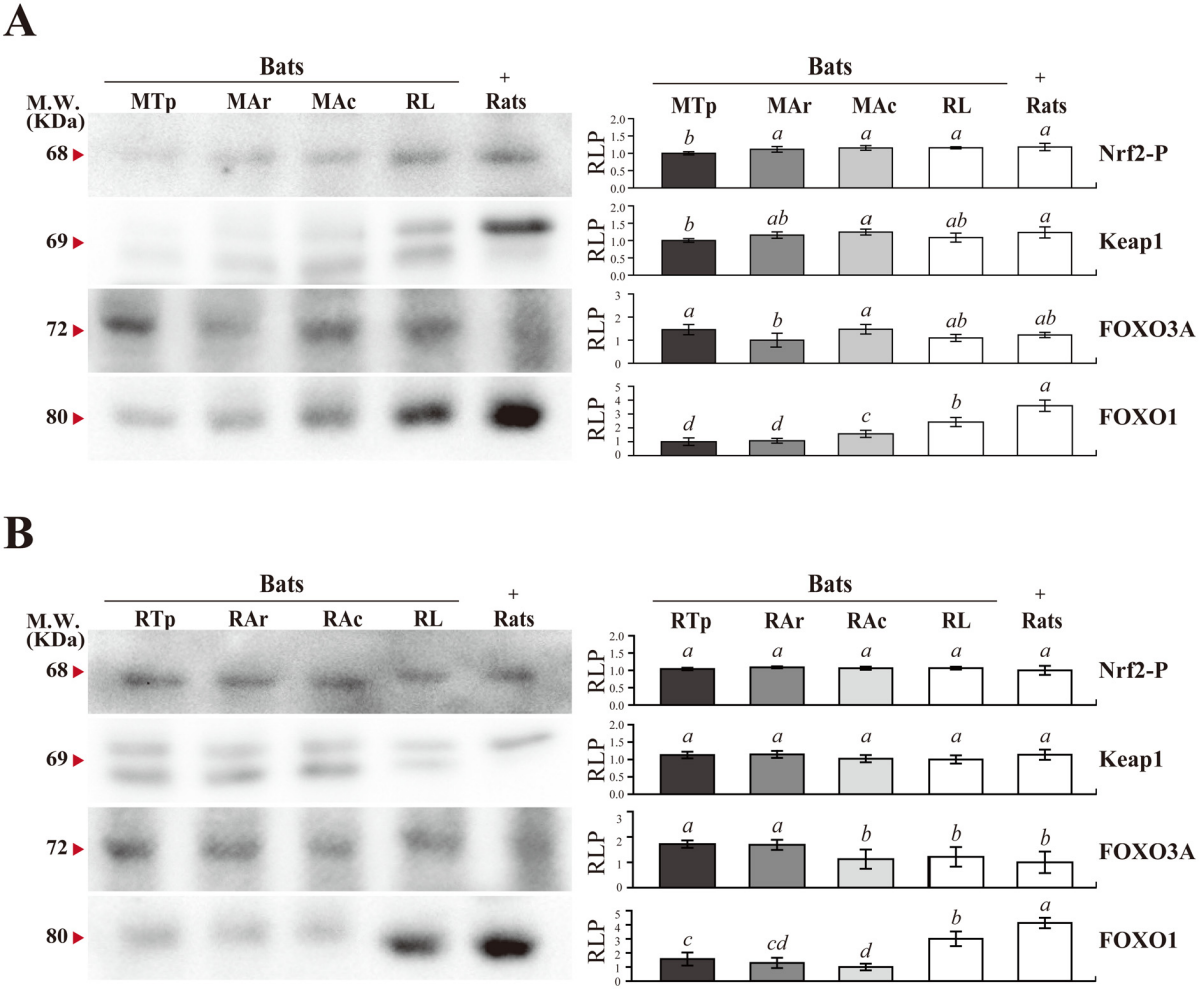
Expression pattern of Nrf2-P, Keap1, FOXO3A, and FOXO1 in bat brain. The amounts of Nrf2-P, Keap1, FOXO3A, and FOXO1 in the brains of the following were determined by Western blotting: non-hibernating *R*. *leschenaultia* bats (RL), rats, **(A)** *M*. *ricketti* bats at torpor (MTp), arousal (MAr), and active states (MAc), and **(B)** *R*. *ferrumequinum* bats at torpor (RTp), arousal (RAr), and active states (RAc). The bands of rats are used as a positive control (+) for antibody reactions. Arrowheads indicate the predicted molecular weight (KDa) of a protein. Relative levels of protein expressions (RLP) are represented by bar plots. The lowest level of a detectable protein is set as 1. Values of group means are represented by italicized letters and are in the order of *a* > *b* > *ab* > *c* > *cd* > *d*. The difference in value is significant (*P* < 0.05) between different letters.

To explore the regulation of antioxidant response during bat hibernation, the relationship among antioxidant proteins, GSH, MDA, and ROS/RNS in *R*. *ferrumequinum* bats and *M*. *ricketti* bats were analyzed by IPA (S4 Table). The results suggested the presence of a network of transcription factors that regulate antioxidant responses in these bats, and Nrf2 and FOXOs were found to play a central role (S2 Fig). The results of IPA were verified by determining the levels of Nrf2 expression and phosphorylation, as well as the expressions of Keap1 and FOXOs. Although no significant expression changes of Nrf2 were found in both species of hibernating bats at different states (data not shown), the levels of phosphorylated Nrf2 and Keap1 were significantly increased in *M*. *ricketti* bats after arousal (Fig 5A). In addition, the levels of FOXO3A in *M*. *ricketti* bats were found to be decreased upon arousal, whereas the levels of FOXO1 were increased at the active state (Fig 5A). No significant changes in the levels of phosphorylated Nrf2 or Keap1 were seen in *R*. *ferrumequinum* bats. The expressions of FOXO3A and FOXO1 were decreased in *R*. *ferrumequinum* bats after arousal (Fig 5B). In addition, two isoforms of Nrf2 and Keap1 were detected by Western blotting as previously described [52].

## Discussion

Both species of hibernating bats (*R*. *ferrumequinum* and *M*. *ricketti*) investigated in this study were found to have a significantly lower level of ROS/RNS in the brain at arousal than at torpor or active state (Fig 1), suggesting that some ROS/RNS in the brains of these bats are removed upon arousal. Previous studies have shown that oxygen consumption in squirrels at arousal is 3 fold higher than that at active and 36 fold higher than that at torpid state [53]. In bats, oxygen use at arousal is 8.7 fold higher than that at active state [14]. Since the rate of ROS/RNS synthesis is correlated positively with oxygen consumption [8], ROS/RNS levels in bat brain should be higher at arousal than at torpid or active state; our results are contrary to this hypothesis (Fig 1). It is possible that excessive ROS/RNS is quickly removed upon arousal in order to protect the brain from oxidative stress (Fig 1). This postulations is supported by the observations that catalase and superoxide dismutase-like activities in Syrian hamsters (*Mesocricetus auratus*) are increased upon arousal [54, 55]. Since torpid bats have an extremely low rate of metabolism, it is assumed that they have a very low level of ROS/RNS in the brain. However, our results showed similar levels of ROS/RNS in the brains of torpid and active bats (Fig 1). A possible explanation for this observation is that torpid bats require basal levels of ROS/RNS to maintain their redox homeostasis.

ROS/RNS levels in the brains of hibernating and non-hibernating bats, rats, and mice were in the range of 7 to 11 X 10^2^ nmol/g, but there were huge variations in levels among individual non-hibernating *R*. *leschenaultia* bats (Fig 1). As *R*. *leschenaultia* bats are very sensitive to human disturbance, it is conceivable that ROS/RNS levels varied widely among individual *R*. *leschenaultia* bats due to differences in their emotional response [56]. It has been postulated that reduced levels of ROS/RNS correlate with the longevity of hibernating bats [44, 48]. Our results are not consistent with this postulation as none of the hibernating bats was found to have a lower level of ROS/RNS than non-hibernating mammals (Fig 1). These results, however, agree with previous reports that the level of ROS/RNS is not the sole determinant of mammalian longevity [44, 48]. Although it is suggested that mammalian longevity is associated positively with a lower production of H_2_O_2_ in heart mitochondria [57], other factors, such as, metabolic rate, oxygen consumption, reproductive rate, and caloric restriction also affect longevity [43].

ROS/RNS could be both deleterious and beneficial. Overproduction of ROS/RNS results in redox imbalance leading to oxidative damages, but a low or moderate level of ROS/RNS is required to serve as second messengers in cell signaling [58]. Hydrogen peroxide signaling has been shown to be involved in the growth and maintenance of neural stem cells in the subgranular zone of the hippocampus and subventricular zone of the lateral ventricles [59]. As mammals maintain their ability to respond to periodic arousal and external stimulations during torpor and arousal cycles [15] and keep their long-term memory after hibernation [11], we speculate that ROS/RNS in torpid bats act as neuromodulators for brain activity rather than stress molecules for oxidative damages. Although ROS/RNS levels were lower in arousal than in torpid or active bats (Fig 1), the levels were similar among arousal bats and non-hibernating bats (Fig 1). These results, together with the finding of a significantly lower MDA level, an index of oxidative damage of lipids, in hibernating bats (*M*. *ricketti* and *R*. *ferrumequinum*) than in non-hibernating bats (*R*. *leschenaultia* and *C*. *sphinx*) (Fig 2), suggest that bats maintain a basal and functional level of ROS/RNS that does not harm the brain during torpor.

The production and decomposition of ROS/RNS in bat brain during different hibernation states remain to be investigated; however, we found that *M*. *ricketti* bats upregulated the expression of most antioxidant proteins (SOD1, SOD2, GSR, GPX1, CAT, NQO1, DJ-1, and TRX2) after arousal (Fig 3 and S1 Fig). In *R*. *ferrumequinum* bats, most of these proteins remained expressed in all three different states (Fig 3 and S1 Fig), and the levels of SOD2, GSR, and TRX2 in *R*. *ferrumequinum* bats were higher than those in *M*. *ricketti* bats at torpor and arousal (Fig 3 and S1 Fig). The total GSH levels were higher in *M*. *ricketti* than in *R*. *ferrumequinum* bats (Fig 4A), but the levels in *M*. *ricketti* bats were not elevated until active state. GSH levels in *R*. *ferrumequinum* bats were significantly elevated after torpor (Fig 4A). These data indicate that *M*. *ricketti* bats use mainly antioxidant proteins and that *R*. *ferrumequinum* bats rely more on LMWAs (i.e., GSH) to cope with oxidative stresses during arousal. In addition, non-hibernating *R*. *leschenaultia* bats had the lowest expression of antioxidant proteins, except GPX1 and TRX2, compared to both species of hibernating bats (Fig 3). Since non-hibernating bats had GSH levels similar to those of hibernating bats (Fig 4A), their low expression of antioxidant proteins may cause insufficient ROS/RNS scavenging leading to oxidative damage. This could explain the higher MDA levels in non-hibernating than in hibernating bats (Fig 2). Rats are not hibernators, but their brain MDA levels were low (Fig 2), similar to those of hibernating bats. This could be due to their production of high amounts of antioxidant proteins (Fig 3).

When cells are exposed to reactive species, the reduced form of glutathione (GSH) is converted to the disulfide form (GSSG). As GSSG accumulates, the GSSG/GSH ratio will increase. A higher GSSG/GSH ratio reflects a more oxidized environment of cells. In this study, hibernating bats, especially *M*. *ricketti*, expressed relatively high levels of antioxidant proteins (Fig 3) and GSH (Fig 4A). Hibernating bats also had limited variation in GSSG/GSH ratio at torpid and active states (Fig 4B), but huge variation in GSSG/GSH ratio at arousal (Fig 4B). Because the regulation of redox metabolism requires energy and nutrition supply, the variation in the expression of various antioxidants among different species of mammals may be due to their diverse feeding habits and metabolic status [22]. The non-hibernating bats examined in this study are fruit bats that obtain a large amount of antioxidant vitamins, minerals, and sugars from foods. Hibernating bats are mostly insectivores, but *M*. *ricketti* bats can also eat fish [60]. The large variation in GSSG/GSH ratio in arousal bats may be a result of dramatic change in metabolic demand. The variation in GSSG/GSH ratio in non-hibernating species (e.g., fruit bats and rodents) may be due to differences in dietary intake (Fig 4B) and emotional responses. Some LMWAs, such as ascorbate, are associated with GSH reduction and have been shown to be oxidized or elevated in levels during arousal of ground squirrels and hamsters [16, 61]. Although bats are progressively losing their ability to produce ascorbate [62], ascorbate may still play a significant role in their antioxidant response [63, 64]. The roles of other LMWAs (e.g., α- tocopherol and urate) and antioxidant related proteins (e.g., heat shock proteins) in antioxidant defense of bats remain to be investigated.

The expression patterns of antioxidants in the two hibernating species of bats are different (Figs 3 and 4). Results of IPA analyses showed that many transcription factors cross talk to regulate the overall antioxidant defense. Among them, NFE2L2 (Nrf2) and FOXO3 (*P* value of overlap < 1 X 10^−10^) appear to play central roles in the regulatory network (S2 Fig). During arousal, the amounts of phosphorylated Nrf2, Keap1, and FOXO1 were increased in *M*. *ricketti* bats (Fig 5A) and were unchanged or decreased in *R*. *ferrumequinum* bats (Fig 5B). However, a lower expression of FOXO3A was found in arousal *M*. *ricketti* bats. This could be due to the activation of PI3K/Akt kinase that increases Nrf2 activity [65] but also enhances proteasome degradation of FOXOs [66]. In ground squirrels, the expression of Nrf2 is increased in heart, liver, and brown adipose tissues but decreased in the brain during hibernation [5, 67], and that of FOXO3A is upregulated at torpor [68]. These observations support the view that Nrf2 and FOXO are major mediators of antioxidant response.

In this study, two distantly related hibernating species of bats had a similar ROS/RNS level (Fig 1) but a significantly lower MDA level (Fig 2) than non-hibernating bats. These findings are consistent with previous reports that the amounts of ROS/RNS do not necessarily represent the degree of oxidative damage [58, 59]. A normal redox status can only be achieved when there is a balance between oxidant production and antioxidant defense. Both enzymatic and non-enzymatic reactions take place cooperatively to eliminate ROS/RNS. Our results indicated that bats have evolved strategies to overcome oxidative insults during hibernation (Figs 3, 4A, 5, and S1 Fig). It has been hypothesized that the homeostasis of ROS/RNS is related to aging and longevity [69]. We found that both hibernating species of bats, which have an exceptional longevity (S1 Table), had limited variation in GSSG/GSH ratio, compared to non-hibernating mammals (Fig 4B). This observation suggests that mammals that have the ability to maintain a relatively stable redox status may have a longer lifespan.

## Conclusion

Our results provide the first evidence that hibernating bats alter their antioxidant defense system in the brain against oxidative insults during arousal. *M*. *ricketti* bats adjust mainly the expression of antioxidant proteins and *R*. *ferrumequinum* bats use both enzymatic and non-enzymatic (e.g., GSH) antioxidants (Figs 3, 4A, and S2 Fig). In general, bat hibernators have higher levels of antioxidant proteins in their active state compared to non-hibernators (Fig 3). Nrf2 and FOXOs signals are important in the regulation of antioxidant defense during bat hibernation (Fig 5 and S1 Fig). Our study supports the hypothesis that different organisms use different strategies to cope with oxidative stress (S3 Fig) [22].

## Supporting Information

S1 Fig Antioxidants in the brains of hibernating bats. In cells, superoxide anions (O_2_∙^-^) are mostly generated by the mitochondrial electron transport chain (ETC), NADPH oxidase (NOX), and endoplasmic reticulum. A leaked electron reacts with oxygen to form O_2_∙^-^. Other reactive species (denoted in red), such as hydroxyl radical (HO∙), hydrogen peroxide (H_2_O_2_), nitric oxide (NO∙), and peroxynitrite anion (ONOO∙^-^) are generated by multiple reactions. Glutathione is presented as oval. The expression levels of each antioxidant protein in different bats at different states are represented by a group of small rectangle boxes that are colored according to the expression levels shown in Fig 3. The small boxes in each group represent the following (from left to right): torpid (MTp), arousal (MAr), and active (MAc) *M*. *ricketti* bats, and torpid (RTp), arousal (RAr), and active (RAc) *R*. *ferrumequinum* bats. Arrows indicate directions of reactions. NQO1: NADPH quinone oxidoreductase 1; SOD1: superoxide dismutase 1; SOD2: superoxide dismutase 2; CAT: catalase; PRDX1: peroxiredoxin 1; PRDX3: peroxiredoxin 3; DJ-1: Parkinson disease protein 7; TRX2: thioredoxin 2; GSR: glutathione reductase; GPX1: glutathione peroxidase 1; -red: reduced state; -ox: oxidative state; -SNO: *S*-nitrosylation.
(TIF)

S2 Fig IPA analyses of regulatory networks. **(A)** Arousal (left panel) or active state (right panel) vs. torpor of *M*. *ricketti* bats. **(B)** Arousal (left panel) or active state (right panel) vs. torpor of *R*. *ferrumequinum* bats. All *P* values of overlap are < 10^−6^. Asterisk (*) indicates *P* value of overlap < 10^−10^.
(TIF)

S3 Fig Species-specific regulation of antioxidant defenses in the brains of hibernating bats. (TIF)

S1 Table Longevity of the mammalian species. (DOCX)

S2 Table Basic information of animals used in this study. (DOCX)

S3 Table Antibodies used in this study. (DOCX)

S4 Table Fold changes of the molecules for IPA analyses. (DOCX)

S5 Table Expression levels (mean ± SD) of the proteins in different mammalian species. (DOCX)
