# Supplementary figures and images for: Antioxidant Defenses in the Brains of Bats during Hibernation
Source: PLoS One. 2016 Mar 24;11(3):e0152135. doi: 10.1371/journal.pone.0152135 (PMC4806925; doi:10.1371/journal.pone.0152135)

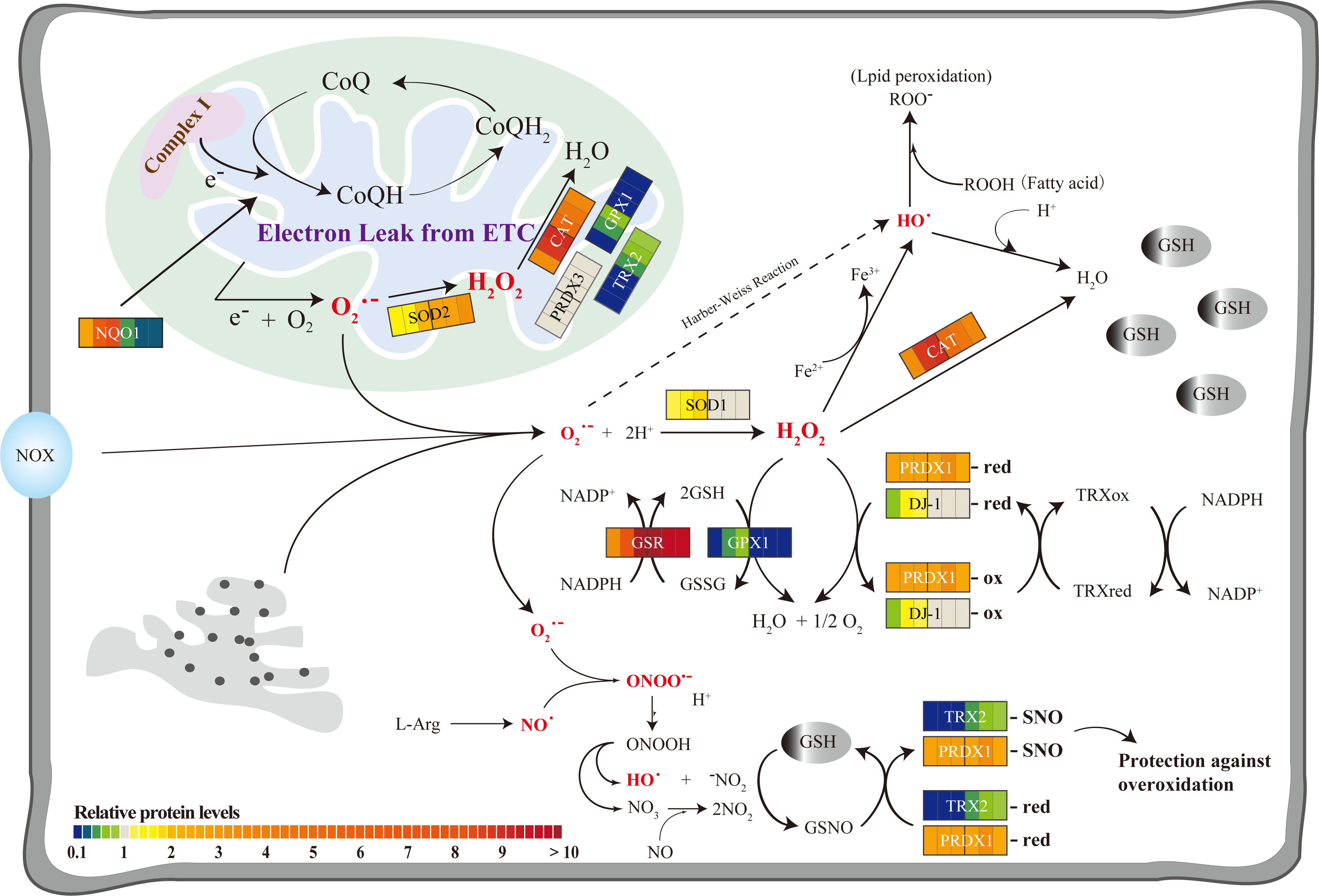

Supplement: S1 Fig — In cells, superoxide anions (O2∙-) are mostly generated by the mitochondrial electron transport chain (ETC), NADPH oxidase (NOX), and endoplasmic reticulum. A leaked electron reacts with oxygen to form O2∙-. Other reactive species (denoted in red), such as hydroxyl radical (HO∙), hydrogen peroxide (H2O2), nitric oxide (NO∙), and peroxynitrite anion (ONOO∙-) are generated by multiple reactions. Glutathione is presented as oval. The expression levels of each antioxidant protein in different bats at different states are represented by a group of small rectangle boxes that are colored according to the expression levels shown in Fig 3. The small boxes in each group represent the following (from left to right): torpid (MTp), arousal (MAr), and active (MAc) M. ricketti bats, and torpid (RTp), arousal (RAr), and active (RAc) R. ferrumequinum bats. Arrows indicate directions of reactions. NQO1: NADPH quinone oxidoreductase 1; SOD1: superoxide dismutase 1; SOD2: superoxide dismutase 2; CAT: catalase; PRDX1: peroxiredoxin 1; PRDX3: peroxiredoxin 3; DJ-1: Parkinson disease protein 7; TRX2: thioredoxin 2; GSR: glutathione reductase; GPX1: glutathione peroxidase 1; -red: reduced state; -ox: oxidative state; -SNO: S-nitrosylation. (TIF) [file pone.0152135.s001.tif]

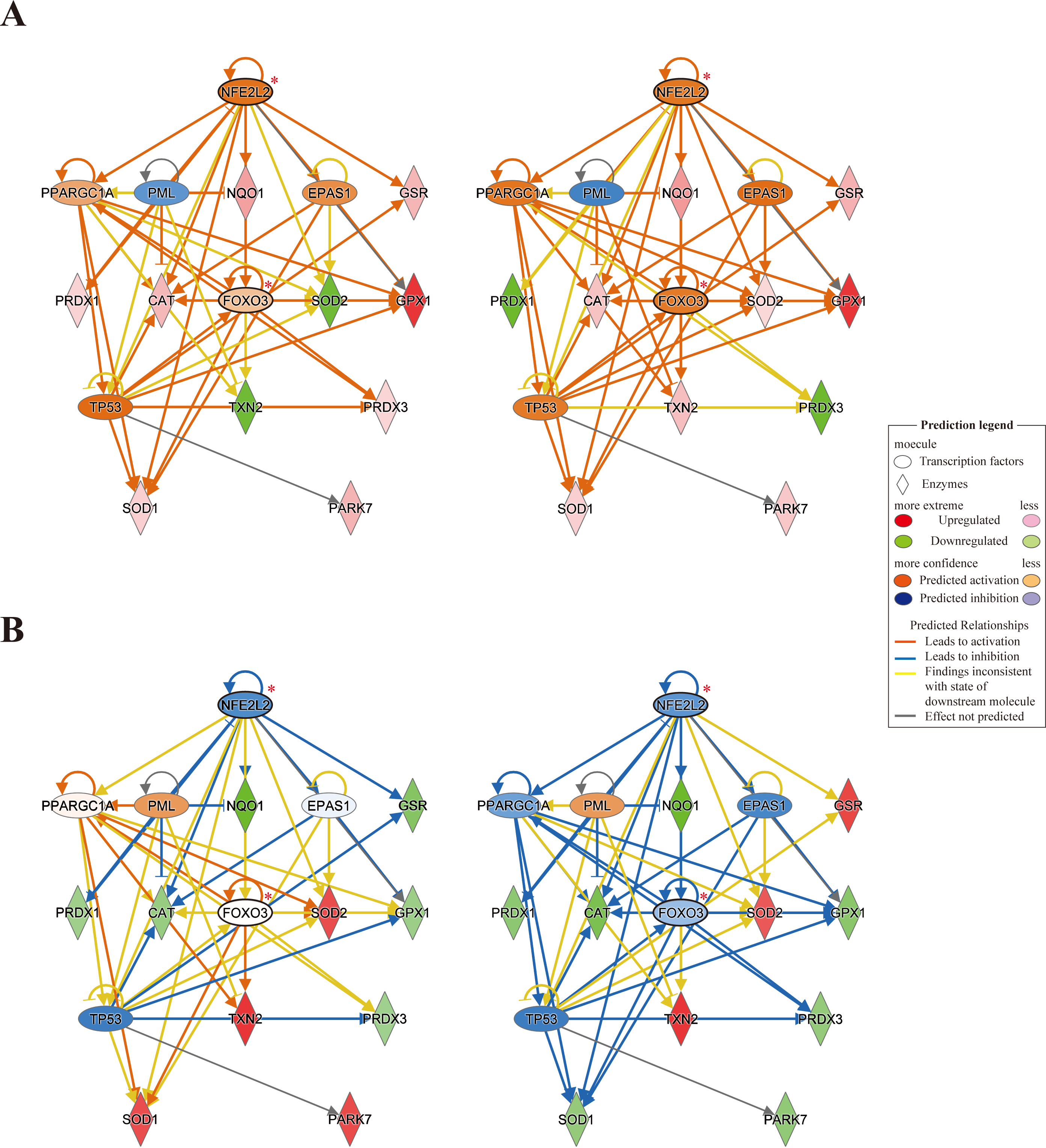

Supplement: S2 Fig — (A) Arousal (left panel) or active state (right panel) vs. torpor of M. ricketti bats. (B) Arousal (left panel) or active state (right panel) vs. torpor of R. ferrumequinum bats. All P values of overlap are < 10−6. Asterisk (*) indicates P value of overlap < 10−10. (TIF) [file pone.0152135.s002.tif]

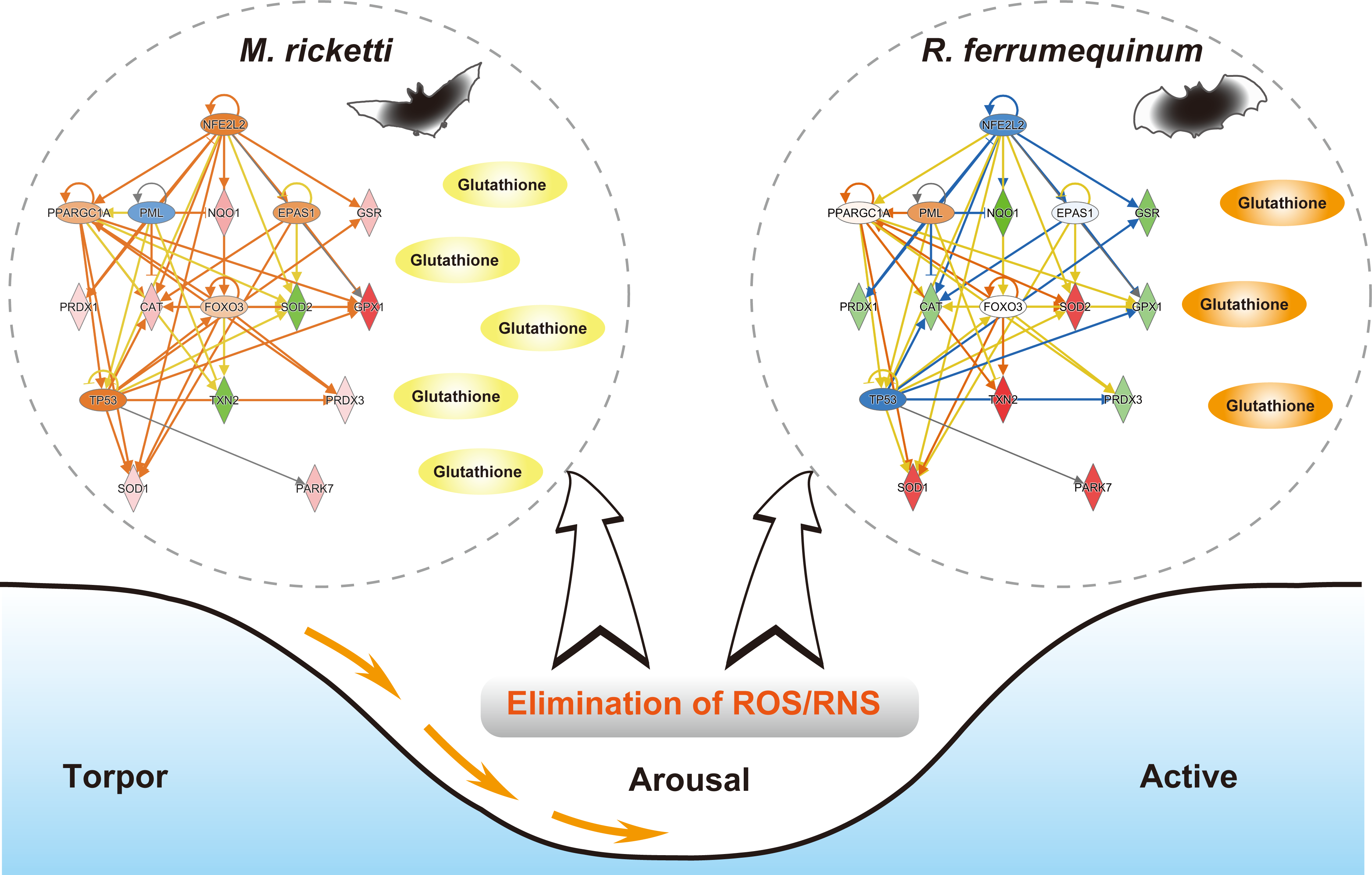

Supplement: S3 Fig — (TIF) [file pone.0152135.s003.tif]
