# Supplementary material for: Antioxidant Defenses in the Brains of Bats during Hibernation
Source: PLoS One. 2016 Mar 24;11(3):e0152135. doi: 10.1371/journal.pone.0152135 (PMC4806925; doi:10.1371/journal.pone.0152135)
Supplement: S1 Table — (DOCX) [file pone.0152135.s004.docx]

**S1 Table. Longevity of the mammalian species**

| **Species** | **Body mass**  **(g)** | **Predicted maximum lifespan (MLSP)**  **MLSP = 3.34**  **(Mass in g)^0.193^** | **Longest longevity**  **(year)** | **Longevity quotient**  **LQ = Actual longevity/MLSP** | **References** |
| --- | --- | --- | --- | --- | --- |
| *Myotis rickitti****^a, b^*** | 22 | 6.06 | 30 | 4.95 | [1] |
| *Myotis lucifugus****^a^*** | 10 | 5.21 | 34 | 6.53 | [2] |
| *Myotis brandti****^a^*** | 7 | 4.86 | 41 | 8.43 | [3] |
| *Rhinolophus ferrumequinum****^a, b^*** | 24 | 6.17 | 30.5 | 4.95 | [4, 5] |
| *Rousettus leschenaulti****^b, c^*** | 90 | 7.96 | 14 | 1.75 | [4, 6] |
| *Cynopterus sphinx****^b, c^*** | 75 | 7.68 | 10 | 1.3 | [4] |
| Mice*^b^* | 25 | 6.22 | 4 | 0.64 | [7] |
| Rats*^b^* | 250 | 9.70 | 4.2 | 0.43 | [6] |

***^a^***Hibernating species of bats

***^b^***Mammalian species used in this study

***^c^***Non-hibernating species of bats

1. Ma J, Zhang L-b, Liang B, Shen J-x, Zhang S-y, Jones G. Piscivorous habit and echolocation sound of Myotis ricketti at Fangshan, Beijing. Zoological Research. 2003;24(4):265-8.

2. Wilkinson GS, South JM. Life history, ecology and longevity in bats. Aging cell. 2002;1(2):124-31.

3. Podlutsky AJ, Khritankov AM, Ovodov ND, Austad SN. A new field record for bat longevity. The Journals of Gerontology Series A: Biological Sciences and Medical Sciences. 2005;60(11):1366-8.

4. Singaravelan N, Marimuthu G. Nectar feeding and pollen carrying from Ceiba pentandra by pteropodid bats. Journal of Mammalogy. 2004;85(1):1-7.

5. Carey J, Judge D. Longevity records: life spans of mammals, birds, amphibians, reptiles, and fish. On-line) Max Planck Institute for Demographic Research Accessed June. 2002;13:2005.

6. Weigl R, Jones ML. Longevity of mammals in captivity: from the living collections of the world: a list of mammalian longevity in captivity: E. Schweizerbart'sche; 2005.

7. Miller RA, Harper JM, Dysko RC, Durkee SJ, Austad SN. Longer life spans and delayed maturation in wild-derived mice. Experimental Biology and Medicine. 2002;227(7):500-8.
