# Supplementary material for: Antioxidant Defenses in the Brains of Bats during Hibernation
Source: PLoS One. 2016 Mar 24;11(3):e0152135. doi: 10.1371/journal.pone.0152135 (PMC4806925; doi:10.1371/journal.pone.0152135)
Supplement: S2 Table — (DOCX) [file pone.0152135.s005.docx]

**S2 Table. Basic information of animals used in this study**

| **Species** | **Status** | **Body mass (g)** | **Rectal temperature (^o^C)** | **Fasting blood glucose concentrations (mmol/L)** |
| --- | --- | --- | --- | --- |
| *Myotis rickitti* | Torpor |  | 8-13 | < 2 |
|  | 2h after arousal | 15-17 | 30-32 | ~ 4.2 |
|  | 24h after arousal |  | 35-37 | ~ 4.5 |
| *Rhinolophus ferrumequinum* | Torpor |  | 8-13 | < 2 |
|  | 2h after arousal | 19-22 | 30-32 | ~ 4.3 |
|  | 24h after arousal |  | 35-37 | ~ 4.8 |
| *Rousettus leschenaulti* | Non-hibernation | 72-83 | 35-36 | ~ 6.85 |
| *Cynopterus sphinx* | Non-hibernation | 39-48 | 35-37 | ~ 4.5 |
| Mice | Non-hibernation | 34-38 | 35-37 | ~ 5.5 |
| Rats | Non-hibernation | 264-257 | 35-37 | ~ 4 |
