# Supplementary material for: Antioxidant Defenses in the Brains of Bats during Hibernation
Source: PLoS One. 2016 Mar 24;11(3):e0152135. doi: 10.1371/journal.pone.0152135 (PMC4806925; doi:10.1371/journal.pone.0152135)
Supplement: S3 Table — (DOCX) [file pone.0152135.s006.docx]

**S3 Table. Antibodies used in this study**

| **Protein Name** | **Product No.** | **Company** | **Polyclonal or Monoclonal** | **Dilution** | **Sample loading/ lane** |
| --- | --- | --- | --- | --- | --- |
| PRDX1 | ab41906 | Abcam Corp. | Polyclonal | 1:5000 | 20 μg |
| PRDX3 | ab86084 | Abcam Corp. | Polyclonal | 1:5000 | 20 μg |
| DJ-1 (PARK7) | ab18257 | Abcam Corp. | Polyclonal | 1:10000 | 20 μg |
| GPX1 | ab22604 | Abcam Corp. | Polyclonal | 1:200 | 20 μg |
| Nrf2 (phospho S40) | ab76026 | Abcam Corp. | Monoclonal | 1:5000 | 40 μg |
| Nrf2 | sc-722 | Santa Cruz Biotechnology, Inc. | Polyclonal | 1:800 | 40 μg |
| SOD2 | sc-30080 | Santa Cruz Biotechnology, Inc. | Polyclonal | 1:10000 | 20 μg |
| NQO1 | sc-16464 | Santa Cruz Biotechnology, Inc. | Polyclonal | 1:400 | 20 μg |
| GSR | sc-32886 | Santa Cruz Biotechnology, Inc. | Polyclonal | 1:1000 | 40 μg |
| KEAP1 | sc-15246 | Santa Cruz Biotechnology, Inc. | Polyclonal | 1:500 | 20 μg |
| FKHR (FOXO1) | sc-9808 | Santa Cruz Biotechnology, Inc. | Polyclonal | 1:500 | 40 μg |
| SOD1 | 10269-1-AP | Proteintech Group, Inc. | Polyclonal | 1:1000 | 20 μg |
| CAT | 21260-1-AP | Proteintech Group, Inc. | Polyclonal | 1:1000 | 20 μg |
| TRX2 | 13089-1-AP | Proteintech Group, Inc. | Polyclonal | 1:1000 | 20 μg |
| FOXO3A | 10849-1-AP | Proteintech Group, Inc. | Polyclonal | 1:1000 | 40 μg |
