# Supplementary material for: Antioxidant Defenses in the Brains of Bats during Hibernation
Source: PLoS One. 2016 Mar 24;11(3):e0152135. doi: 10.1371/journal.pone.0152135 (PMC4806925; doi:10.1371/journal.pone.0152135)
Supplement: S4 Table — (DOCX) [file pone.0152135.s007.docx]

**S4 Table. Fold changes of the molecules for IPA analyses**

| **ID*^a^*** | **MA/T*^b^*** | **MC/T*^b^*** | **RA/T***^c^* | **RC/T*^c^*** |
| --- | --- | --- | --- | --- |
| **Q06830** | 1.13076 | -1.0378 | -1.0473 | -1.0912 |
| **P30048** | 1.03225 | -1.0086 | -1.0293 | -1.0184 |
| **Q99497** | 1.93023 | 1.68405 | 1.0471 | -1.0562 |
| **P00441** | 1.18485 | 1.43681 | 1.03211 | -1.0013 |
| **P04179** | -1.0335 | 1.17401 | 1.04389 | 1.16352 |
| **P04040** | 1.80059 | 1.86551 | -1.1633 | -1.4021 |
| **P15559** | 2.46533 | 3.17181 | -2.2372 | -1.8412 |
| **Q99757** | -1.0386 | 1.95012 | 1.18398 | 1.45548 |
| **P00390** | 1.86232 | 2.37895 | -1.4868 | 1.33182 |
| **P07203** | 5.54769 | 6.73067 | -1.0846 | -1.1475 |
| **C00051** | 1 | 1.1 | 1.1 | 1.15 |
| **D0008** | -1.125 | 1.08 | 1.05 | 1 |
| **D00074** | -1.125 | 1.08 | 1.05 | 1 |
| **C19440** | 1 | 1 | 1.08 | 1 |

*^a^*The UniProt number of proteins or KEGG number of chemical compounds.

*^b^*MA/T or MC/T: the amount of molecules in *M. ricketti* bats at arousal or active state versus that at torpor.

*^c^*RA/T or RC/T: the amount of molecules in *R. ferrumequinum* bats at arousal or active state versus that at torpor.
