# Supplementary material for: Antioxidant Defenses in the Brains of Bats during Hibernation
Source: PLoS One. 2016 Mar 24;11(3):e0152135. doi: 10.1371/journal.pone.0152135 (PMC4806925; doi:10.1371/journal.pone.0152135)
Supplement: S5 Table — (DOCX) [file pone.0152135.s008.docx]

**S5 Table. Expression levels (mean ± SD) of the proteins in different mammalian species**

| **Proteins** | **MT*^a^*** | **MA*^a^*** | **MC*^a^*** | **RL*^b^*** | **Rat** | **RT*^c^*** | **RA*^c^*** | **RC*^c^*** |
| --- | --- | --- | --- | --- | --- | --- | --- | --- |
| SOD1 | 1.81±0.06 | 2.15±0.21 | 2.61±0.53 | 1.43±0.28 | 1.43±0.30 | 1.50±0.34 | 1.55±0.34 | 1.50±0.18 |
| SOD2 | 2.38±0.18 | 2.31±0.10 | 2.80±0.33 | 1.34±0.27 | 3.16±0.64 | 3.49±0.34 | 3.64±0.38 | 4.06±0.54 |
| GSR | 10.10±2.07 | 18.82±12.35 | 24.04±11.49 | 4.46±3.32 | 35.22±21.10 | 34.26±22.26 | 23.04±15S.12 | 45.63±37.44 |
| GPX1 | 2.05±1.01 | 11.39±3.01 | 13.82±4.39 | 20.25±6.53 | 17.71±4.18 | 2.49±0.89 | 2.30±0.15 | 2.17±0.41 |
| CAT | 37.35±3.87 | 65.44±10.62 | 64.7±39.73 | 6.00±5.41 | 16.69±5.76 | 31.51±10.29 | 27.09±6.28 | 22.47±9.96 |
| NQO1 | 109.11±26.8 | 268.99±20.79 | 346.07±83.2 | 41.63±22.5 | 78.45±38.50 | 20.87±13.14 | 9.33±12.77 | 11.34±6.98 |
| DJ-1 | 1.46±0.41 | 2.81±0.24 | 2.45±0.13 | 1.99±0.32 | 3.13±0.42 | 2.27±0.19 | 2.38±0.18 | 2.15±0.37 |
| TRX2 | 2.28±0.71 | 2.19±0.44 | 4.44±1.53 | 26.87±9.03 | 15.31±7.55 | 15.64±10.07 | 18.51±10.09 | 22.76±15.56 |
| PRDX1 | 3.82±0.25 | 4.31±0.44 | 4.16±0.52 | 1.47±0.33 | 3.26±0.44 | 3.88±0.23 | 3.70±0.41 | 3.55±0.74 |
| PRDX3 | 1.21±0.12 | 1.25±0.11 | 1.24±0.04 | 1.20±0.07 | 1.26±0.06 | 1.23±0.03 | 1.20±0.05 | 1.21±0.09 |

*^a^*MT, MA, and MC represent *M. ricketti* bats in torpor, arousal, and active states, respectively.

*^b^*RL: *R. leschenaultia* bats.

*^c^*RT, RA, and RC represent *R. ferrumequinum* bats in torpor, arousal, and active states, respectively.
